# Trichloroethylene and its metabolite TaClo lead to degeneration of substantia nigra dopaminergic neurones: Effects in wild type and human A30P mutant α-synuclein mice

**DOI:** 10.1016/j.neulet.2019.134437

**Published:** 2019-10-15

**Authors:** P.C. Keane, P.S. Hanson, L. Patterson, P.G. Blain, P. Hepplewhite, A.A. Khundakar, S.J. Judge, P.J. Kahle, F.E.N. LeBeau, C.M. Morris

**Affiliations:** a Medical Toxicology Centre, Wolfson Building, Newcastle University, Claremont Place, Newcastle-Upon Tyne, NE2 4AA, UK; b Institute for Neuroscience, Newcastle University, Cookson Building, Framlington Place, Newcastle upon Tyne, NE2 4HH, UK; c Alzheimer’s Society Doctoral Training Centre, Newcastle University, Edwardson Building, Newcastle upon Tyne, NE4 5PL, UK; d German Center for Neurodegenerative Diseases (DZNE) Tübingen, Hertie Institute for Clinical Brain Research, Laboratory of Functional Neurogenetics, University of TÜbingen, 72076 Tübingen, Germany

**Keywords:** PD, Parkinson’s disease, TCE, trichloroethylene, TaClo, 1-trichloromethyl-1,2,3,4-tetrahydro-β-carboline, DA, dopaminergic, SNpc, substantia nigra pars compacta, DMSO, dimethylsulfoxide, MPTP, 1-methy,4-phenyl, 1,2,3,6 tetrahyrdopyridine, CE, coefficient of error, TH, tyrosine hydroxylase, ANOVA, analysis of variance, Trichloroethylene, TaClo, Parkinson’s disease, α-Synuclein, Neurodegeneration

## Abstract

Parkinson’s disease (PD) is characterised pathologically by degeneration of the dopaminergic (DA) neurones of the substantia nigra pars compacta (SNpc) and the presence of α-synuclein containing Lewy body inclusions. Trichloroethylene (TCE) has been suggested as a potential environmental chemical that may contribute to the development of PD, via conversion to the neurotoxin, 1-Trichloromethyl-1,2,3,4-tetrahydro-β-carboline (TaClo). We investigated the effect of an 8 week exposure to TCE or TaClo on wild type and, as an experimental model of PD, A30P mutant α-synuclein overexpressing mice using a combination of behaviour and pathology. TCE or TaClo exposure caused significant DA neuronal loss within the SNpc in both wild type and transgenic mice. Cell numbers were lower in A30P animals than wild type, however, no additive effect of TCE or TaClo exposure and A30P overexpression was found. TCE or TaClo did not appear to lead to acceleration of motor or cognitive deficits in either wild type or A30P mutant mice, potentially because of the modest reductions of DA neuronal number in the SNpc. Our results do however suggest that TCE exposure could be a possible factor in development of PD like changes following exposure.

## Highlights

• Parkinson’s disease (PD) may have an environmental component involving toxin exposure.
• Trichloroethylene (TCE) is a major environmental contaminant and can convert to the toxin TaClo.
• We administered TCE and TaClo to wild type and alpha-synuclein mutant mice as a model of PD.
• TCE and TaClo caused substantia nigra neurone loss but alpha-synuclein mutation was not additive.

## 1 Introduction

Parkinson’s disease (PD) is the leading cause of neurodegenerative motor impairment and is characterised by progressive loss of the dopaminergic (DA) neurones of the substantia nigra pars compacta (SNpc). Clinically, PD manifests as a triad of bradykinesia, rigidity and resting tremor that becomes apparent when 50–70% of the DA neurones of the SNpc are lost [1]. The exact mechanism by which SNpc cell death occurs is poorly understood, but is thought to involve both environmental and genetic factors. One current suggestion is that in genetically predisposed individuals, exposure to an unidentified environmental chemical(s) leads to cell death in the enteric nervous system and spread of alpha-synuclein pathology to the central nervous system and the SNpc [2]. Trichloroethylene (TCE), a commonly used industrial solvent, is one potential environmental chemical that may contribute to the development of PD and similar forms of parkinsonism. TCE can be continually present in the environment in areas of previous TCE use and workers can be exposed to levels of 100 ppm in the air [3]. There have been links between chronic exposure to TCE and parkinsonian symptoms [[4], [5], [6], [7]], and treatment of rats or mice with TCE causes SNpc DA neurone degeneration [8,9]. TCE can be converted, via chloral, to a potential neurotoxin, 1-trichloromethyl-1,2,3,4-tetrahydro-β-carboline (TaClo) in man [10], which can cross the blood brain barrier [11]. TaClo can cause up to 50% cell death in primary DA neurones cultures [12], as well as *in vivo* exposure, leading to a reduction in DA metabolism [13] and altered locomotor activity [[14], [15], [16]]. How TCE or TaClo might lead to parkinsonism is unclear since both potentially can cause peripheral neuronal damage and consequent pathological spread, or may directly affect SNpc neuronal viability.

Alpha-synuclein, the major constituent of Lewy bodies in PD, has been shown to be important in the development of experimental PD [17] and mutations in the α-synuclein gene cause familial PD [18]. One such mutation (A30P) [19], has been shown to reduce α-synuclein degradation [22,23], potentially leading to increased cellular α-synuclein levels. The A30P mutation also causes increased oligomerisation and fibrillization into toxic aggregates [20]. C57BL/6 mice expressing human A30P α-synuclein develop symptoms that replicate aspects of PD with abnormal age dependent α-synuclein accumulation in the brainstem, thalamus, and cortex, along with abnormal locomotor behaviour and are a useful model of PD [21].

While there is evidence for both genetic and environmental risk factors in development of PD, for most cases there is likely a complex interplay between genetic and environmental influences in the causation of PD. We therefore tested the hypothesis that exposure to TCE might contribute to PD development, and whether TaClo might achieve similar effects. We therefore assessed the effects of TCE and TaClo exposure in A30P mutant α-synuclein mice as a model of PD and in wild type mice.

## 2 Materials & methods

All experiments were carried out according to the UK Animals (Scientific Procedures) Act of 1986, with UK Home Office Guidance and the European Community Council Directive of 24 November 1986 (86/609/EEC).

A30P mutant human α-synuclein overexpressing mice develop a motor defect at 14–16 months of age [[21], [22], [23]]. To determine if TCE or TaClo exposure caused an acceleration of any deficits, exposure experiments were conducted on younger mice. Fifteen week old male and female wild type C57BL/6 mice (Charles River UK, Margate) and A30P mutant human α-synuclein overexpressing C57BL/6 mice were housed in clean holding rooms. Mice were kept in groups of up to 6 in solid bottomed, saw dust filled filter top individually ventilated cages with environmental enrichment available throughout the study period. The rooms were illuminated by fluorescent lights set to give a 12 h light-dark cycle (on 07.00, off 19.00). Animals were fed a diet of RM03 rodent diet (Special Diet Services, Edinburgh, UK) *ad libitum* and had free access to mains tap water.

Ten wild type and ten A30P overexpressing mice per treatment group (five male and five female) were intraperitoneally injected twice weekly with 10 ml/kg of either olive oil as vehicle, 1000 mg/kg TCE in olive oil or 2 mg/kg TaClo in 0.1% DMSO in 0.9% NaCl for eight weeks. Doses were established using prior dose ranging studies in wild type mice as being suitable and showing no acute short term effects on body weight and feeding. Each mouse was weighed prior to every injection during the dosing period and then weekly for the duration of the study. The effects of chemical administration on weight loss were analysed by ANOVA followed by Bonferroni test with significance set at *p* <  0.05.

### 2.1 Behavioural testing

Pre-dose, and at 4, 8, 12, 16, 20, 24, 28, 32, 36 and 40 weeks after initial treatment, all animals were subjected to rotarod, grip-strength and pole tests. For the Barnes maze, spatial learning was tested in all animals at 41 weeks post initial dose and spatial memory in week 41 (short-term) and 42 (long-term). The effects of chemical exposure on behaviour were analysed by ANOVA followed by Bonferroni test with genotype, sex, and time as cofactors.

Motor function was assessed by an accelerating rotarod (IITC Life Sciences WPI, Hertfordshire, UK) [24]. Animals were trained at a constant speed of 3 rpm prior to testing. On test days, animals were tested in 3 trials with 15 min between trials. For each trial, animals were placed on the rod at a speed of 3 rpm and the rod steadily accelerated to 30 rpm over 300 s and the fall latency for each animal recorded. If they did not fall, a latency of 300 s was recorded.

Fore paw grip strength was measured using a grip test device (BIOSEB, Vitrolles, France)[25]. Each mouse was allowed to grip a wire mesh with its forepaws and was gently pulled back by the tail until its grip was broken. The average of three measurements was taken for each animal.

Fine motor function was assessed using the pole test [26]. Animals were trained to grip, face up, on the top of an 8 mm diameter, 55 cm high polypropylene threaded pole (Shop4fasteners.co.uk, Sheffield, UK), within the home cage and had to turn around and climb down on 3 separate occasions prior to testing. On test days, animals were timed in the activity for 3 trials separated by at least 15 min. Time to turn, time to descend, total time to complete the trial and number of falls over 3 trials were recorded. If the animal did not complete the task or fell, a time of 30 s was recorded.

Spatial learning and memory were assessed using a Barnes maze [27] in weeks 40 and 41. The paradigm consisted of a 96 cm diameter grey plastic disk maze with 20 equally spaced holes (5 cm diameter), 2 cm from the maze edge at 70 cm above the floor. One hole, the ‘target’, had access to a small dark escape chamber located under the maze, with no visual discrimination between this and other holes. A barrier surrounded the maze with distinct visual cues (A4 size filled square, circle, triangle and cross) attached at the cardinal points to give the animals spatial reference points and the room, including the experimenter, were kept in constant configuration throughout the duration of the experimental protocol. All experimental procedures were recorded using Panasonic SDR-S26 video camera for scoring.

On Day 0 (adaption phase) mice were placed in a 10.5 cm diameter chamber in the centre of the maze (start chamber) for 10 s before being released. The mouse was then gently guided to the target hole and encouraged to enter the escape chamber. Once in the escape chamber, the entrance was covered and the mouse was left for 2 min then returned to the home cage. Four times per day on days 1–4 with a 15 min inter-trial interval mice were placed in the start chamber for 10 s and then released into the maze and they were allowed to explore for 3 min to assess spatial acquisition. During the exploration period, primary errors (the amount of times incorrect holes were explored before finding the target – nose pokes), total errors (the amount of times incorrect holes explored before entering the target hole escape chamber), primary latency (the amount of time taken to find target hole) and total latency (the amount of time taken to enter target hole) were recorded. The trial ended when the mouse entered the escape chamber or 180 s had elapsed if the escape chamber was not entered, and the mouse was gently encouraged to enter the chamber. Once in the escape chamber, the entrance was covered and the mouse was left for 1 min then returned to the home cage. Between trials the maze was cleaned with 70% ethanol and the maze rotated 90° to remove olfactory cues. The effects of chemical exposure on spatial memory were analysed by Two-way Repeated Measures ANOVA followed by Bonferroni test with significance at *p* <  0.05.

On Day 5, 24 h following the last training day, and on Day 12 (probe trials), a trial was conducted to assess short and long term retention respectively. The escape chamber was removed and mice were placed in the start chamber as previously and then released into the maze to explore for 90 s. Number of pokes (errors) in each numbered hole was recorded. The effects of chemical exposure on short and long term spatial memory were analysed by unpaired *t-*test for each hole. Significance was set at *p* <  0.05.

### 2.2 Neuronal counting

Animals were terminally anaesthetised in 3% isofluorane (Isoflo® 100% w/w Inhalation Vapour, Abbott Laboratories Ltd, Maidenhead, UK) in O_2_ and killed by rapid decapitation at 42 weeks post dose, just prior to typical onset of motor symptoms. Brains were dissected from animals, hemisected and fixed by immersion in ice cold 10% formaldehyde (Sigma) in phosphate buffered saline. Brain tissue from 6 animals (3 male and 3 female) were randomly selected from each treatment group and embedded in paraffin wax. Coronal tissue sections (30 μm) were taken rostral to the SNpc at bregma -2.78 mm throughout the SNpc until caudal to bregma -3.78 mm and every 5th section selected for analysis, giving a total of 9 sections per animal. Sections were stained for DA neurones with anti-tyrosine hydroxylase antibody and general nuclei stained using Cresyl Violet. Briefly, sections were de-waxed in xylene (Fisher Scientific) before rehydration in decreasing ethanol solutions. Antigen retrieval was carried out by boiling in 1.27 mM EDTA (Tetrasodium Dihydrate, USB Products, High Wycombe, UK) pH 8 in a microwave for 10 min before allowing to cool for 20 min and then immediately transferring to ddH_2_O. Sections were quenched in 0.9% H_2_O_2_ in TBS followed by 3 washes in TBS and then blocked in goat serum in TBS for 30 min prior to primary (rabbit polyclonal antibody to TH (Abcam) 1:500 in TBS containing blocking goat serum at 4 °C overnight) and secondary incubation (Vectastain Elite ABC Rabbit IgG kit, Vector Laboratories, Peterborough, UK) 1:200 in blocking serum in TBS for 30 min at room temperature. Sections were washed then treated with Vectastain ABC reagent for 30 min at room temperature, washed and then exposed to 0.25 mg/ml 3,3′-Diaminobenzidine (DAB) in TBS with 0.02% H_2_O_2_ for 10 min, before being washed and counterstained with Cresyl Violet in Acetate Buffer (pH 4.5), dehydrated in ethanol, cleared in xylene and coverslips mounted with DPX (Fisher Scientific).

Unbiased estimates of the total number of TH positive neurones were calculated using a Zeiss Imager.Z1 microscope attached to an AxiocamMR3c camera with StereologerTM Ver 2.1 CP-Version (Stereology Resource Centre, Chester, Maryland, USA) software. A frame height of 12 μm ± a guard height of 2 μm were used based on an average section thickness following shrinkage during processing of 16.56 ± 0.31 μm and there was no significant difference between thickness in any treatment group (see Supplementary Table 1).

Total number and volume of TH positive neurones were counted at high (x40) magnification using unbiased multi-level (fraction-based) estimates throughout the SNpc. Volumes of all TH positive neurones were estimated using an isotropic uniform randomly assigned nucleator probe [28]. The coefficient of error (CE) for cell number (num) and volume (vol) estimates were calculated [29] and CE values < 0.10 were considered acceptable (see Supplementary Table 1).

Alpha-synuclein immunohistochemistry was used to determine if Lewy body like alpha-synuclein inclusions were formed in the SNpc following TCE or TaClo exposure and used anti-phosphorylated alpha-synuclein antibody (1:100 dilution; Abcam, Ab51253) following antigen retrieval with 0.01 M sodium citrate buffer (pH6.0) for 20 min. Antibody binding was detected using HRP conjugated anti-rabbit antibody (1:200 dilution; Vector, PK82000) and sections developed using DAB as previously.

## 3 Results

Animals in all treatment groups showed no overt signs of toxicity and showed a similar normal increase in weight over time (Supplementary Fig. 1).

### 3.1 Behavioural tests

**Fig. 1:**
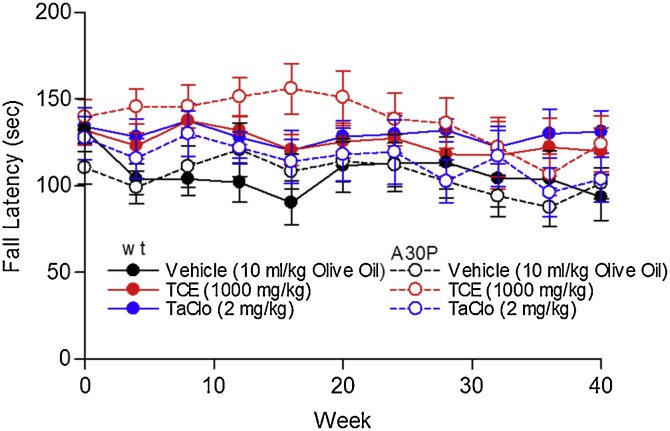
**Effect of TCE and TaClo on A30P α-synuclein and wild type C57BL/6 Mice on Motor Function Using Rotarod.** Average fall latency (sec) of vehicle (10 ml/kg olive oil), TCE (100 mg/kg) or TaClo (2 mg/kg), treated wild type or A30P C57BL/6 mice over time, triplicate trials. Data presented as mean ± SEM (n = 8–10). Significant difference over time (*p* < 0.05, *p* < 0.001 respectively), No significant difference between treatment groups or of treatment over time, Two-Way (treatment, time) Repeated Measures ANOVA.

To assess motor function, an accelerating rotarod was used prior to and every 4 weeks until 40 weeks after the first dose. No significant differences between treatment groups and no significant effect of treatment over time was found in either A30P mutant or wild type mice, but there was a significant decrease in performance over time across all groups (Fig. 1). No significant difference was seen at any time point for any treatment when compared to vehicle.

**Fig. 2:**
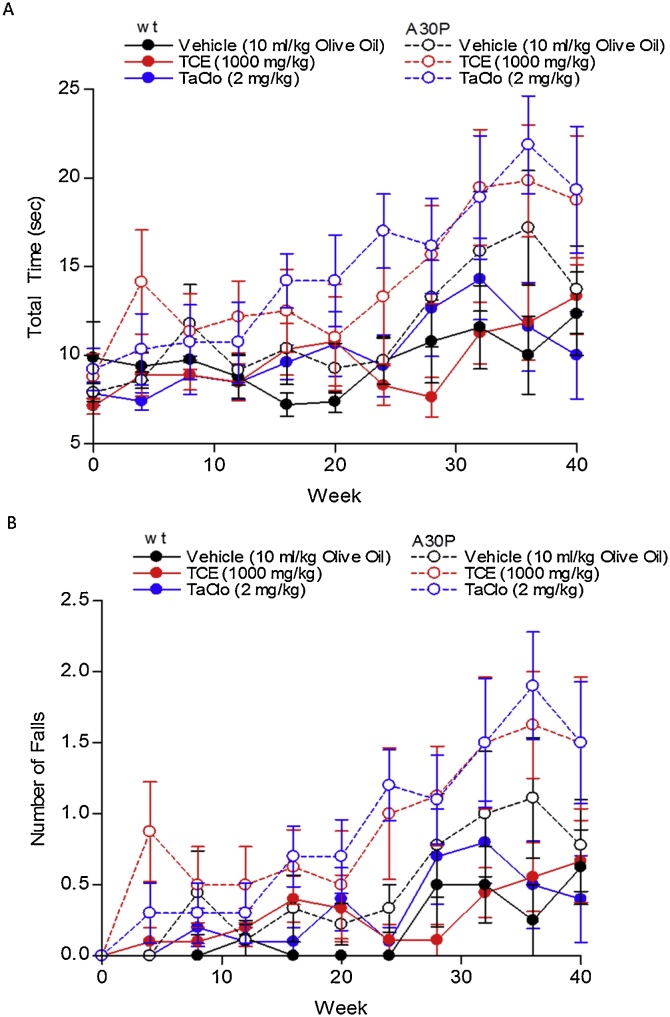
**Effect of TCE and TaClo on wild type and A30P α-synuclein C57BL/6 Mice as Assessed by the Pole Test.** Average (A) total time, and (B) Number of falls per trial of vehicle (10 ml/kg olive oil), TCE (100 mg/kg) or TaClo (2 mg/kg), treated wild type or A30P α-synuclein overexpressing C57BL/6 mice over time, triplicate trials. Data presented as mean ± SEM (n = 8–10 animals per group). A significant effect of time was seen on time to complete the task for all groups (*p* < 0.05). No significant difference between treatment groups or of treatment over time was observed, Two-Way (time, treatment) Repeated Measures ANOVA.

The pole test showed no significant differences in total trial time between treatment groups or effect of treatment over time, but there was a significant increase in total trial time over the test period in all mice indicating poorer performance with age in both groups (Fig. 2). No significant differences in number of falls were seen between treatment groups with no significant effect of treatment over time, but a significant increase in falls was observed in both A30P and wild type mice over time (Fig. 2B). No significant changes in falls in TCE and TaClo treated mice were seen in the A30P group (Fig. 2B).

As abnormalities in grip strength have been reported in PD mice [30] and spinal pathology is seen in A30P mice, grip strength was tested. There were no significant differences observed in grip strength between treatment groups, and no significant effect of treatment over time in any group, although there was a significant difference over time in both groups indicating decreased grip with age (Supplementary Fig. 2).

**Fig. 3:**
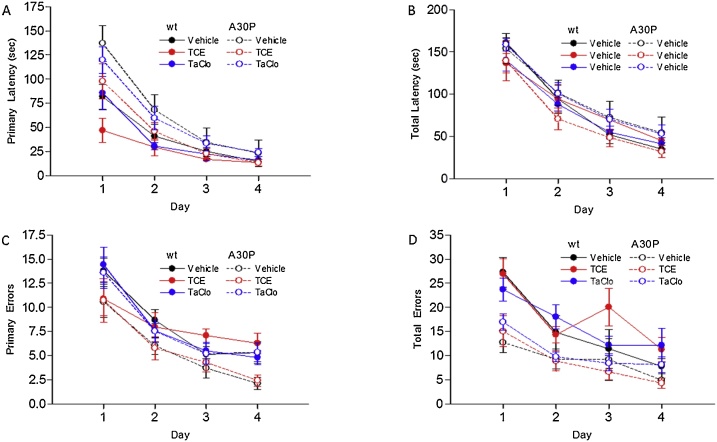
**Effect of TCE and TaClo on wild type and A30P α-synuclein overexpressing C57BL/6 Mouse Spatial Learning Assessed by the Barnes Maze.** Average (A) primary latency, (B) total latency (both sec), (C) primary errors and (D) total errors to find target of vehicle (10 ml/kg olive oil), TCE (100 mg/kg) or TaClo (2 mg/kg), treated wild type or A30P α-synuclein overexpressing C57BL/6 mice over time, quadruplicate trials. Data presented as mean ± SEM (n = 8–10). (A and B) Significant difference over time (*p* < 0.001), No significant difference between treatment groups or of treatment over time, Two-Way Repeated Measures ANOVA.

Previous studies have shown that A30P mice show cognitive deficits [31]. To determine if TCE or TaClo accelerated cognitive changes, learning and memory were assessed using a Barnes maze. For all treatment groups and in A30P and wild type mice, there were no significant differences between treatment groups or of treatment over time, but all animals significantly improved at finding and entering the escape box over the study period (Fig. 3). Memory on Day 5 (short term memory) and Day 12 (long term memory) showed no significant differences in any treatment group and in either A30P or wild type mice (Supplementary Fig. 3).

### 3.2 DA cell number

**Fig. 4:**
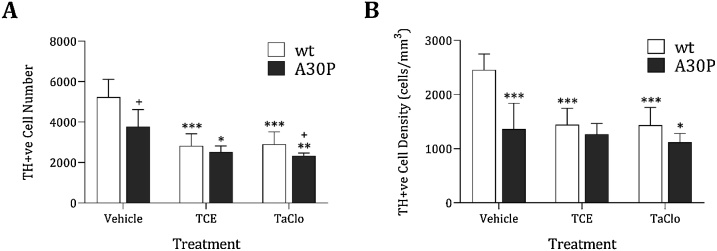
**Effect of TCE and TaClo on SNpc DA Neuron Number and Density in A30P α-synuclein overexpressing and wild type C57BL/6 Mice.** Total (A) number and (B) density of TH-positive cells in the SNpc of TCE (1000 mg/kg), TaClo (2 mg/kg) and vehicle (olive oil) treated wild type (wt) and A30P overexpressing C57BL/6 mice wer assessed using a stereological approach. Analysis of all data and variables showed a significant overall effect *F*(4, 58) = 5.217, *p* < 0.001, Wilks' Lambda=0.425 (MANOVA). *Post hoc* analysis on the outcome variables revealed a significant effect on cell density *F*(5, 29) = 7.361, *p* < 0.003 and cell number *F*(5, 29) = 4.236, *p* = 0.043. Data presented as mean ± SD (n=5/6). ***p < 0.001, **p < 0.01, *p < 0.05 when compared to strain vehicle, +*p* < 0.05 when compared to same treatment wt group (univariate ANOVA). No significant effect was seen on treatment between strains.

As SNpc DA neurone loss is characteristic of PD [1], DA neurone number and size were assessed using stereology to accurately estimate DA neurones. Multivariate analysis of variance (MANOVA) showed a statistically significant interaction effect between mouse model and treatment on the cell number, volume and density *F*(4, 58) = 5.217, *p* < 0.001, Wilks' Lambda=0.425. *Post hoc* univariate analysis on the outcome variables revealed significant effects of strain and treatment on cell density *F*(5, 29) = 7.361, *p* < 0.003 and number *F*(5, 29) = 4.236, *p* = 0.043, but not on cell volume *F*(5, 29) = 0.163, *p* = 0.850. When comparing TH-positive cell (DA neurone) number between A30P and wild type mice, A30P animals showed a significantly lower cell number compared to wild type mice when comparing vehicle treated animals (*p* = 0.014) (Fig. 4A). In wild type animals, a significant decrease in TH-positive cell number in the SNpc was seen in both TCE and TaClo exposed groups (*p* < 0.001; Fig. 4A, and Supplementary Fig. 4), with cells reduced to approximately 50% of vehicle levels in wild type animals (Fig. 4A). For A30P animals, exposure to TCE (*p* =  0.012) and TaClo (*p* = 0.002) caused a significant reduction of TH-positive cell number to approximately 70% of vehicle treated A30P mice (Fig. 4A). TaClo treated A30P mice showed significantly reduced cell TH-positive neurone number when compared to TaClo treated wild type animals (*p* = 0.049, Fig. 4A). Analysing cell density in vehicle only animals showed a significant reduction in cell density in A30P mice compared to wild type mice (*p* = 0.001; Fig. 4B). Following TCE or TaClo treatment, TH-positive cell density in the SNpc showed a 50% decrease in TCE and TaClo treated wild type animals compared to vehicle wild type animals (*p* < 0.001; Fig. 4B). In A30P mice, no reduction in density between vehicle and TaClo (*p* < 0.61) or in TCE treated A30P mice (*p* = 1.00; Fig. 4B) was observed. Estimated SNpc volumes were unchanged (see Supplementary Fig. 5). This data suggests that exposure to both TCE and TaClo leads to a decrease in TH-positive neurones in the SNpc, but that there is only a small additive effect of mutant A30P overexpression in combination with these chemicals. TH-positive neuronal cell body volume was unchanged (Supplementary Fig 6). Use of phosphorylated alpha-synuclein antibody to detect Lewy body like inclusions failed to detect any changes in SNpc in vehicle or treated wild type or A30P overexpressing mice (data not shown).

## 4 Discussion

During this study, wild-type and A30P mutant overexpressing C57BL/6 mice from all groups showed normal weight gain throughout the duration of the study with no significant differences apparent between treatment groups, suggesting that TCE and TaClo were relatively well tolerated.

The rotarod test has been previously used to show a decrease in motor function following acute exposure to MPTP and rotenone [53, 54] and in a rat model of TCE exposure [8]. No significant decrease in motor function was observed in TCE or TaClo mice compared to vehicle treated groups in wild type or A30P mutant mice in the current study. Similarly, we found no difference in motor function using rotorod between wild type and A30P mice, consistent with previous studies [28]. Since we aimed to show acceleration of pathology by TCE or TaClo by determining motor behaviour before the normal onset of major motor symptoms, we can conclude that neither TCE nor TaClo lead to any marked acceleration of major motor symptoms.

Fine motor control is reduced in the pole test and beam test in the A30P mouse [32]. Analysis of mice in this study by the pole test found no difference in fine motor control between vehicle and TCE or TaClo treated groups in either wild type or A30P overexpressing animals, similar to findings with hanging wire [32]. All treatment groups of both strains showed a general increase in the time taken to turn and descend the pole and to complete the task, as well as in the number of falls recorded. This decrease in fine motor control over time may be due to habituation as it occurred across all treatment groups and the majority of previous published uses of the pole test have not typically used it repeatedly.

There was a significant decrease in grip strength over time in all treatment groups of both strains, which manifested as a sharp decrease from pre-dose to 4 weeks, which then levelled off across the rest of the study period. No significant difference following treatment or between strains in wild type or A30P animals was seen. Grip strength may not be a sensitive marker for motor dysfunction since mice that show decreased motor function assessed by rotarod have no significant change in grip strength [33]. The strain of A30P mutant α-synuclein overexpressing mice used shows spinal cord pathology, with a weakening of extremities at 17 months [21,23] and defects in fine motor function [32]. However, no differences in grip strength or gait were seen between wild type and A30P mutant animals in this study, indicating that specific motor tests may be required [32]. TCE and TaClo therefore appear not to cause any marked acceleration of a major motor phenotype in A30P animals.

PD has been widely associated with cognitive decline [34]. In addition, there is a decrease in cognition in A30P mutant α-synuclein overexpressing mice, as well as α-synucleinopathy in the central nucleus of the amygdala, an area involved in cognitive behaviour in mice [31]. In this study, no significant differences were found following treatment in either wild type or A30P mutant animals in any parameter analysed suggesting that TCE or TaClo have no effect on spatial learning. However, when comparing wild type and A30P mutant animals, A30P mutant animals took significantly longer to find the target hole on the first day of the trial, suggesting a possible learning deficit when compared to wild type C57BL/6. Spatial memory was unaffected in TCE or TaClo treated and vehicle groups in short or long term memory in either strain. This lack of effect of TCE and TaClo on spatial learning and memory correlates with a study which reported no effect on learning and memory in TaClo exposed rats using the COGITAT hole board [35]. Combined with our results, this suggests that TCE/TaClo do not contribute to the cognitive deficits seen in PD in toxin-based rodent models.

DA neurone numbers in this study showed a significant decrease in both wild type and A30P mice exposed to TCE and TaClo. This finding is supported by studies that found a similar decrease in DA neurone number in rats acutely exposed to TCE [8]. A30P overexpression led to a significant drop in the SNpc DA neurone numbers in the vehicle and TaClo treated groups, with the effect being more pronounced in the vehicle group. Although a slight decrease in DA neurone number in the SNpc of TCE and TaClo treated A30P animals was seen when compared to wild type, no major additive effect was present. This is consistent with reports that sensitivity to MPTP is unaltered in A30P mice [22] or in wild type human α-synuclein overexpressing mice exposed to paraquat [36]. Density of DA neurones in the SNpc is relatively consistent with the number of neurones in all groups, which is to be expected as there was no significant difference in SNpc volume between treatment groups or strain. Individual DA neuronal volume was measured and although no treatment effect was seen in cell volume in either strain, A30P mutant α-synuclein overexpressing animals showed a trend towards larger cell volumes than wild type in vehicle and TCE groups and were significantly larger in TaClo treated mice.

## 5 Conclusion

The data observed in this study suggests that TCE and TaClo, can cause SNpc DA neurone degeneration, but at the doses used, no significant acceleration of motor deficits in either wild type or A30P overexpressing mice. It is possible that the levels of DA cell death in the SNpc of treated animals are insufficient to cause motor dysfunction since over 70–80% SNpc cell death is required before behavioural deficits are seen [1,37]. While TCE and TaClo exposure led to significant DA neuronal loss no additive effect of chemical exposure and A30P overexpression was found. In conclusion, exposure to TCE or TaClo can cause DA neuronal cell death in the SNpc in vivo, suggesting TCE exposure as a possible contributory factor in development of PD.

## Declaration of Competing Interest

None.

## References

[1] Fearnley J.M., Lees A.J. (1991). Ageing and Parkinson’s disease: substantia nigra regional selectivity. Brain.

[2] Braak H. (2003). Staging of brain pathology related to sporadic Parkinson’s disease. Neurobiol. Aging.

[3] Todd G.D., U.D.o.H.a.H. Services (2019). Toxicological Profile for Trichloroethylene.

[4] Gash D.M. (2008). Trichloroethylene: Parkinsonism and complex 1 mitochondrial neurotoxicity. Ann. Neurol..

[5] Goldman S.M. (2011). Solvent exposures and parkinson disease risk in twins. Ann. Neurol..

[6] Guehl D. (1999). Trichloroethylene and parkinsonism: a human and experimental observation. Eur. J. Neurol..

[7] Reif J.S. (2003). Neurobehavioral effects of exposure to trichloroethylene through a municipal water supply. Environ. Res..

[8] Liu M. (2011). Trichloroethylene induces dopaminergic neurodegeneration in Fisher 344 rats. J. Neurochem..

[9] Liu M. (2018). Trichloroethylene and Parkinson’s disease: risk assessment. Mol. Neurobiol..

[10] Bringmann G. (1999). Identification of the dopaminergic neurotoxin 1-trichloromethyl-1,2, 3,4-tetrahydro-beta-carboline in human blood after intake of the hypnotic chloral hydrate. Anal. Biochem..

[11] Riederer P. (2002). Biochemical and pharmacological characterization of 1-trichloromethyl-1,2,3,4-tetrahydro-beta-carboline: a biologically relevant neurotoxin?. Eur. J. Pharmacol..

[12] Rausch W.D. (1995). Studies of the potentially endogenous toxin TaClo (1-trichloromethyl-1,2,3,4-tetrahydro-beta-carboline) in neuronal and glial cell cultures. J. Neural Transm. Suppl..

[13] Grote C. (1995). Biochemical lesions of the nigrostriatal system by TaClo (1-trichloromethyl-1,2,3,4-tetrahydro-beta-carboline) and derivatives. J. Neural Transm. Suppl..

[14] Sontag K.H. (1995). Long-term behavioural effects of TaClo (1-trichloromethyl-1,2,3,4-tetrahydro-beta-carboline) after subchronic treatment in rats. J. Neural Transm. Suppl..

[15] Heim C., Sontag K.H. (1997). The halogenated tetrahydro-beta-carboline “TaClo”: a progressively-acting neurotoxin. J. Neural Transm. Suppl..

[16] Sontag T.A. (2009). Alterations of nocturnal activity in rats following subchronic oral administration of the neurotoxin 1-trichloromethyl-1,2,3,4-tetrahydro-beta-carboline. J. Neural Transm. (Vienna).

[17] Dauer W. (2002). Resistance of alpha -synuclein null mice to the parkinsonian neurotoxin MPTP. Proc. Natl. Acad. Sci. U. S. A..

[18] Polymeropoulos M.H. (1997). Mutation in the alpha-synuclein gene identified in families with Parkinson’s disease. Science.

[19] Kruger R. (1998). Ala30Pro mutation in the gene encoding alpha-synuclein in Parkinson’s disease. Nat. Genet..

[20] Song W. (2009). The Parkinson disease-associated A30P mutation stabilizes alpha-synuclein against proteasomal degradation triggered by heme oxygenase-1 over-expression in human neuroblastoma cells. J. Neurochem..

[21] Kahle P.J. (2000). Subcellular localization of wild-type and Parkinson’s disease-associated mutant alpha -synuclein in human and transgenic mouse brain. J. Neurosci..

[22] Rathke-Hartlieb S. (2001). Sensitivity to MPTP is not increased in Parkinson’s disease-associated mutant alpha-synuclein transgenic mice. J. Neurochem..

[23] Kahle P.J. (2001). Selective insolubility of alpha-synuclein in human Lewy body diseases is recapitulated in a transgenic mouse model. Am. J. Pathol..

[24] Jones B.J., Roberts D.J. (1968). The quantiative measurement of motor inco-ordination in naive mice using an acelerating rotarod. J. Pharm. Pharmacol..

[25] Cabe P.A. (1978). A simple recording grip strength device. Pharmacol. Biochem. Behav..

[26] Matsuura K. (1997). Pole test is a useful method for evaluating the mouse movement disorder caused by striatal dopamine depletion. J. Neurosci. Methods.

[27] Barnes C.A. (1979). Memory deficits associated with senescence: a neurophysiological and behavioral study in the rat. J. Comp. Physiol. Psychol..

[28] Gundersen H.J. (1988). The new stereological tools: disector, fractionator, nucleator and point sampled intercepts and their use in pathological research and diagnosis. APMIS.

[29] West M.J., Slomianka L., Gundersen H.J. (1991). Unbiased stereological estimation of the total number of neurons in thesubdivisions of the rat hippocampus using the optical fractionator. Anat. Rec..

[30] Colotla V.A. (1990). Effects of MPTP on locomotor activity in mice. Neurotoxicol. Teratol..

[31] Freichel C. (2007). Age-dependent cognitive decline and amygdala pathology in alpha-synuclein transgenic mice. Neurobiol. Aging.

[32] Ekmark-Lewen S. (2018). Early fine motor impairment and behavioral dysfunction in (Thy-1)-h[A30P] alpha-synuclein mice. Brain Behav..

[33] Sedelis M. (2000). MPTP susceptibility in the mouse: behavioral, neurochemical, and histological analysis of gender and strain differences. Behav. Genet..

[34] Lawton M. (2018). Developing and validating Parkinson’s disease subtypes and their motor and cognitive progression. J. Neurol. Neurosurg. Psychiatry.

[35] Sontag T.A. (2007). The long-term effects of the neurotoxin 1-trichloromethyl-1,2,3,4-tetrahydro-beta-carboline (TaClo) on cognitive performance in rats. J. Neural. Transm. Suppl..

[36] Fernagut P.O. (2007). Behavioral and histopathological consequences of paraquat intoxication in mice: effects of alpha-synuclein over-expression. Synapse.

[37] Tillerson J.L. (2002). Detection of behavioral impairments correlated to neurochemical deficits in mice treated with moderate doses of 1-methyl-4-phenyl-1,2,3,6-tetrahydropyridine. Exp. Neurol..

