# Supplementary material for: Trichloroethylene and its metabolite TaClo lead to degeneration of substantia nigra dopaminergic neurones: Effects in wild type and human A30P mutant α-synuclein mice
Source: Neurosci Lett. 2019 Oct 15;711:134437. doi: 10.1016/j.neulet.2019.134437 (PMC6892271; doi:10.1016/j.neulet.2019.134437)
Supplement: Supplementary file 1 [file mmc1.docx]

| Group | Section Thickness (μm) | Cells counted | DA number CE | Cell Volume CE |
| --- | --- | --- | --- | --- |
| **Wild type** | | | | |
| Vehicle | 16.78+0.31 | 258+38 | 0.064 | 0.017 |
| TCE | 16.42+0.24 | 139+33 | 0.087 | 0.018 |
| TaClo | 16.62+0.29 | 144+29 | 0.086 | 0.021 |
| **A30P** | | | | |
| Vehicle | 16.50+0.44 | 188+43 | 0.076 | 0.019 |
| TCE | 16.39+0.21 | 127+15 | 0.090 | 0.014 |
| TaClo | 16.52+0.24 | 117+7 | 0.093 | 0.016 |

**Supplementary Table 1** Grouped average stereological analysis parameters. Section thickness and cells counted presented as mean±SD, CE values presented as mean (n=5/6).


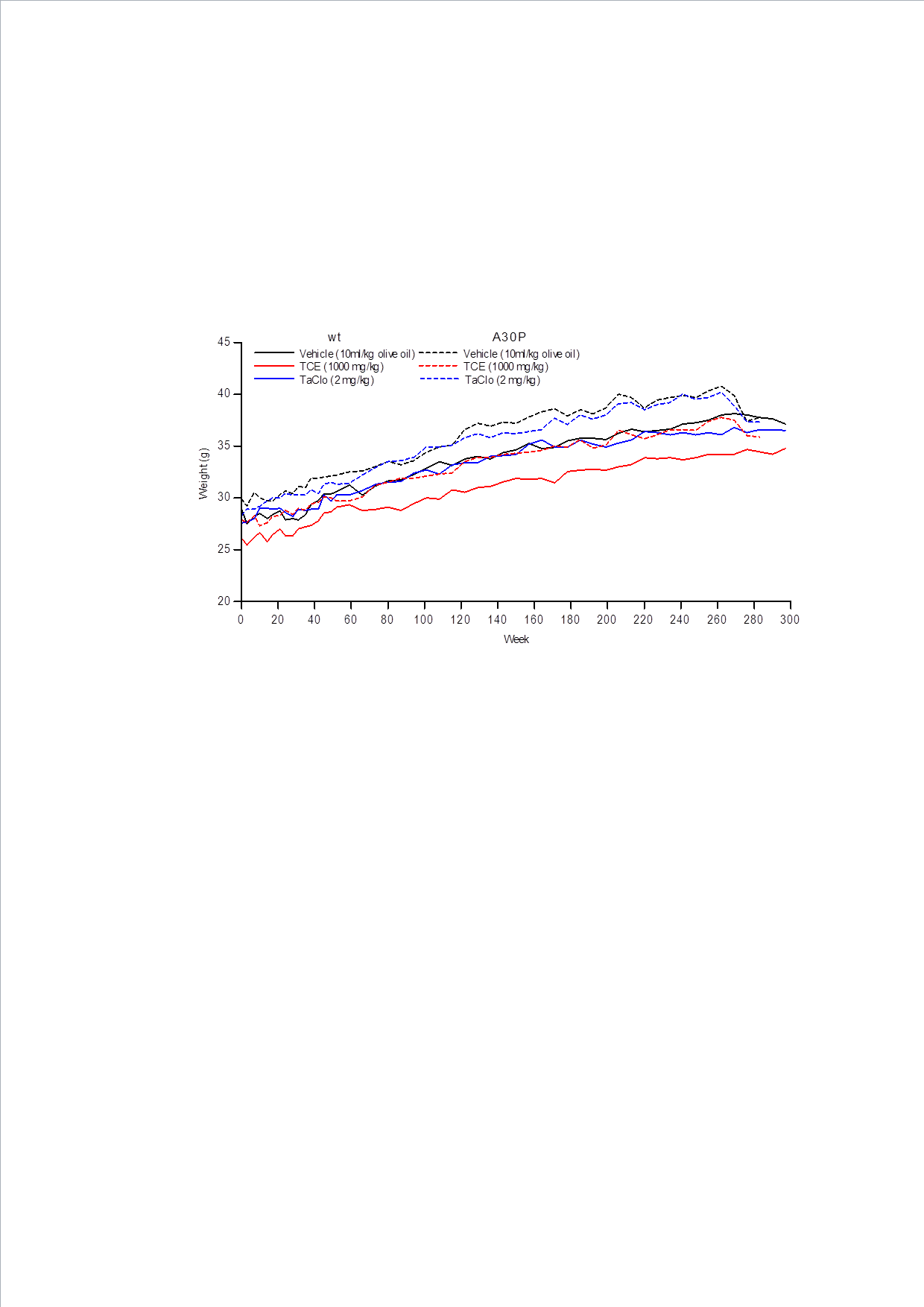


**Supplementary Fig 1: The Effect of TCE and TaClo on A30P α-synuclein overexpressing and wild type C57BL/6 Mice Weights.**

Mice were treated at 15 weeks of age (time 0) with olive oil vehicle, TCE or TaClo for a period of eight weeks. Weight (g) of TCE (1000mg/kg), TaClo (2mg/kg) or control (10ml/kg Olive Oil) treated wild type (wt) or A30P C57BL/6 mice over time. Data presented as mean (n=8-10). Significant difference over time (P<0.001), No significant difference between treatment groups or treatment over time, Two-Way Repeated Measures ANOVA


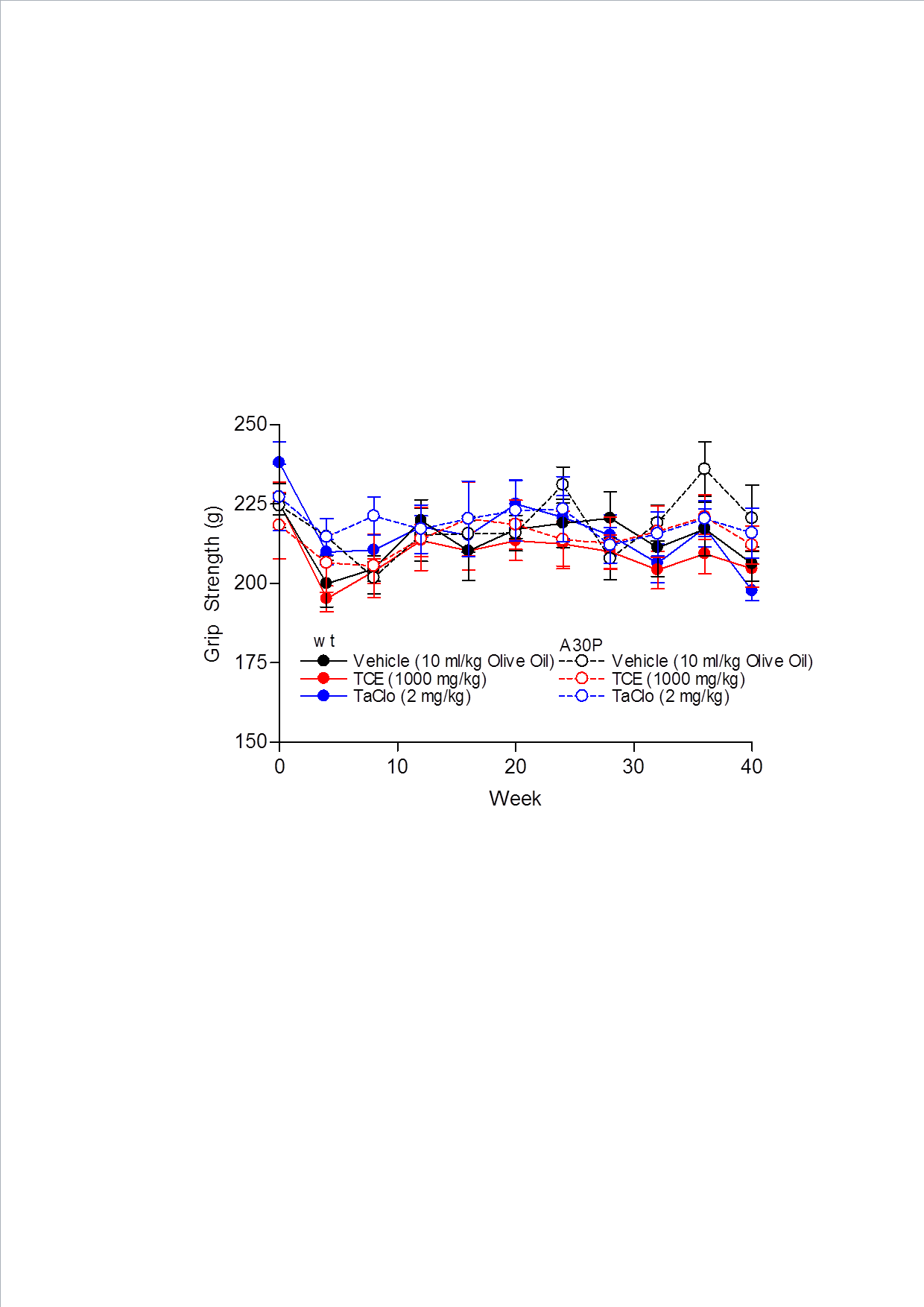


**Supplementary Fig. 2: Effect of TCE and TaClo on A30P α-synuclein overexpressing and wild type C57BL/6 Mouse Forepaw Grip Strength**

Average grip strength (g) of vehicle (10ml/kg olive oil), TCE (100mg/kg) or TaClo (2mg/kg), treated wild type (wt) or A30P C57BL/6 mice over time, triplicate trials. Data presented as mean + SEM (n=8-10 animals per group). Significant difference over time (wild type P<0.001, A30P, P<0.05), No significant difference between treatment groups or of treatment over time, Two-Way Repeated Measures ANOVA.


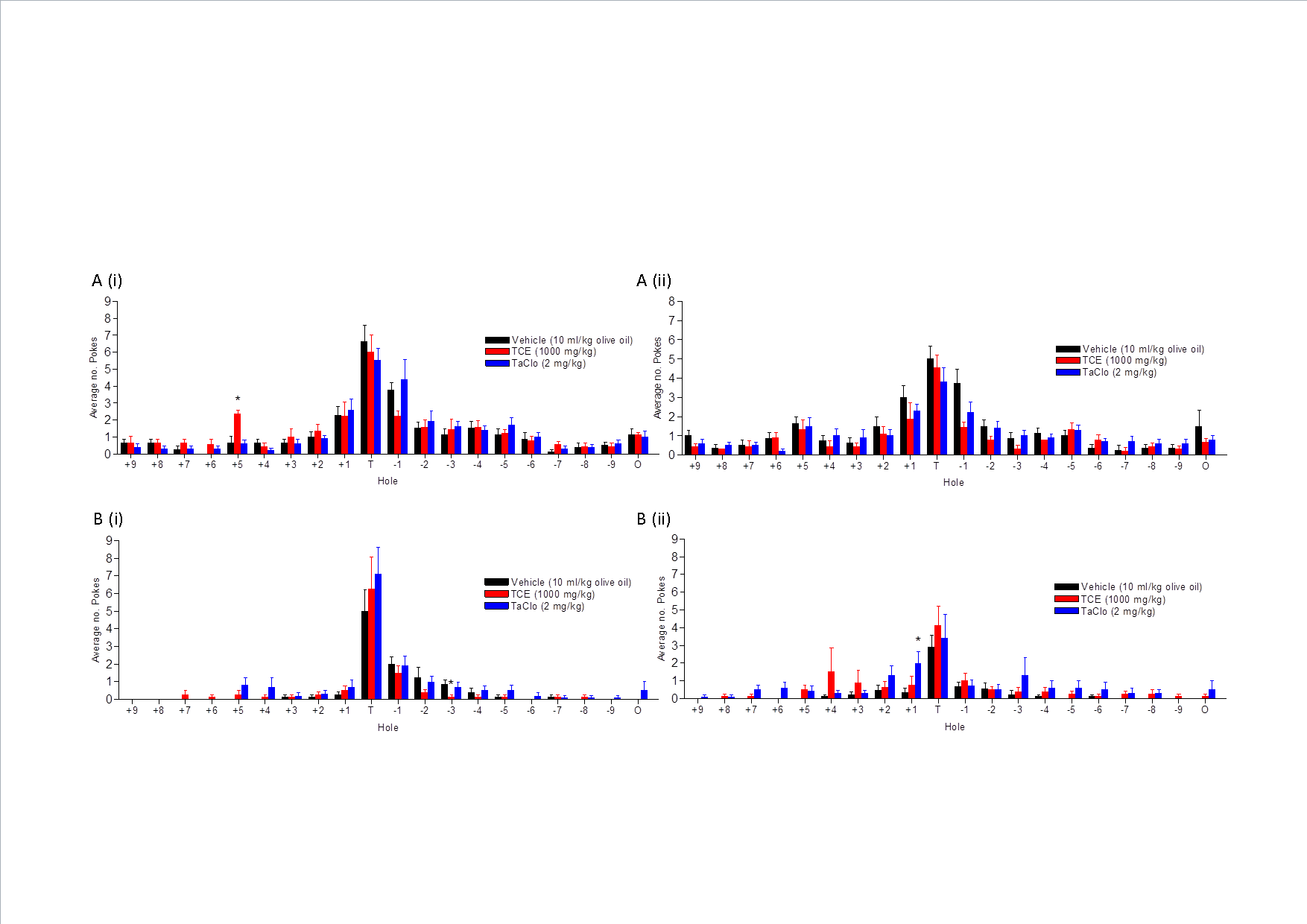


**Supplementary Fig. 3: Effect of TCE and TaClo on A30P α-synuclein overexpressing and wild type C57BL/6 Mouse Spatial Memory Assessed by Barnes Maze Nose Pokes**

Average nose pokes in each hole during (i) short term (Day 5) or (ii) long term (Day 12) probe test of vehicle (10ml/kg olive oil),TCE (100mg/kg) or TaClo (2mg/kg), treated (A) wild type or (B) A30P C57BL/6 mice. Data presented as mean + SEM (n=8-10). *P<0.05 when compared to vehicle (unpaired t-test).


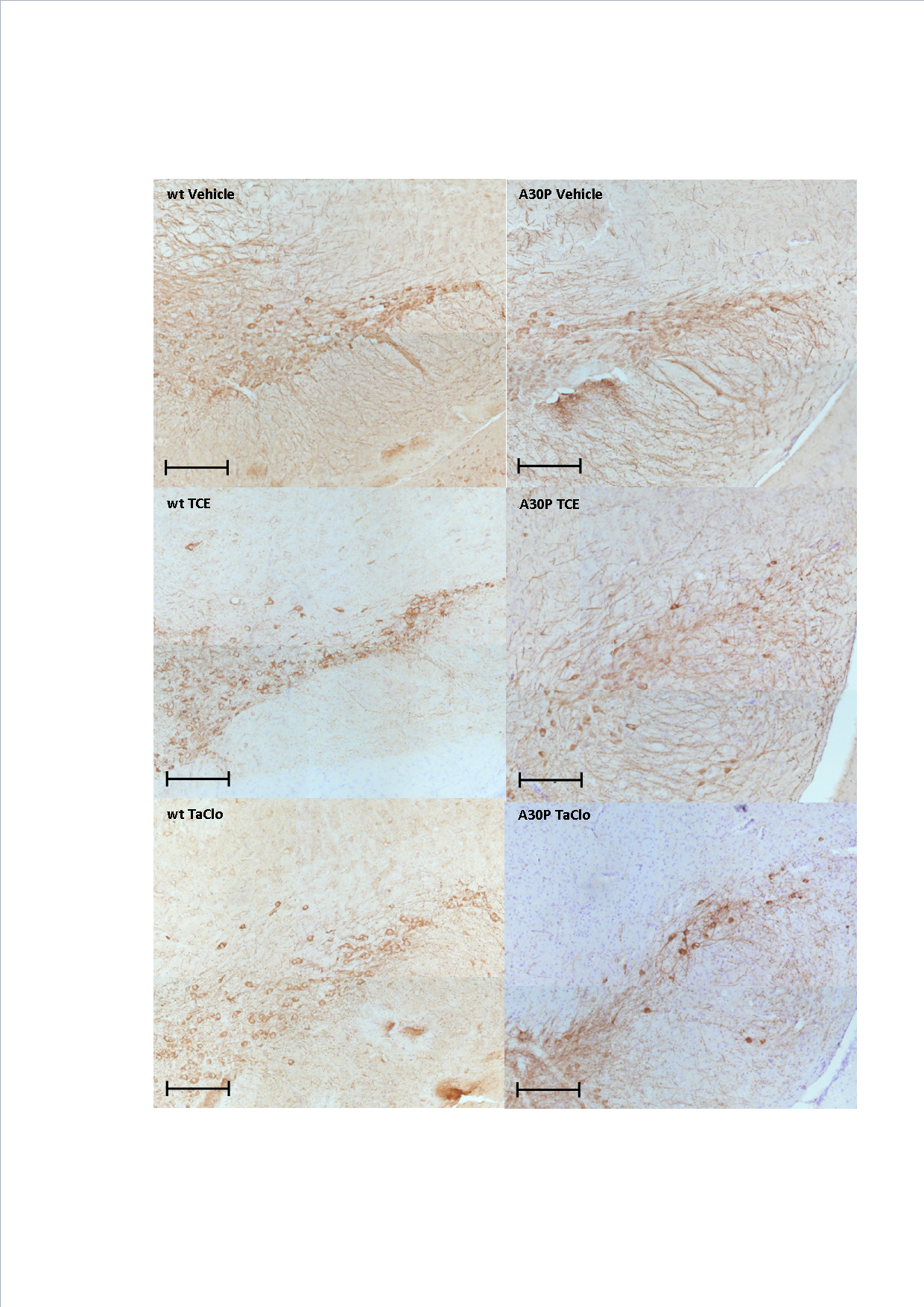


**Supplementary Fig. 4 TH positive staining in TCE & TaClo treated A30P α-synuclein overexpressing and wild type C57BL/6 Mice**

Representative images of TH positive staining in the SNpc in Control, 500mg/kg TCE & 2mg/kg TaClo in or wild type (wt) or A30P α-synuclein overexpressing C57BL/6 (x40 magnification, scale bars represent 200µm).


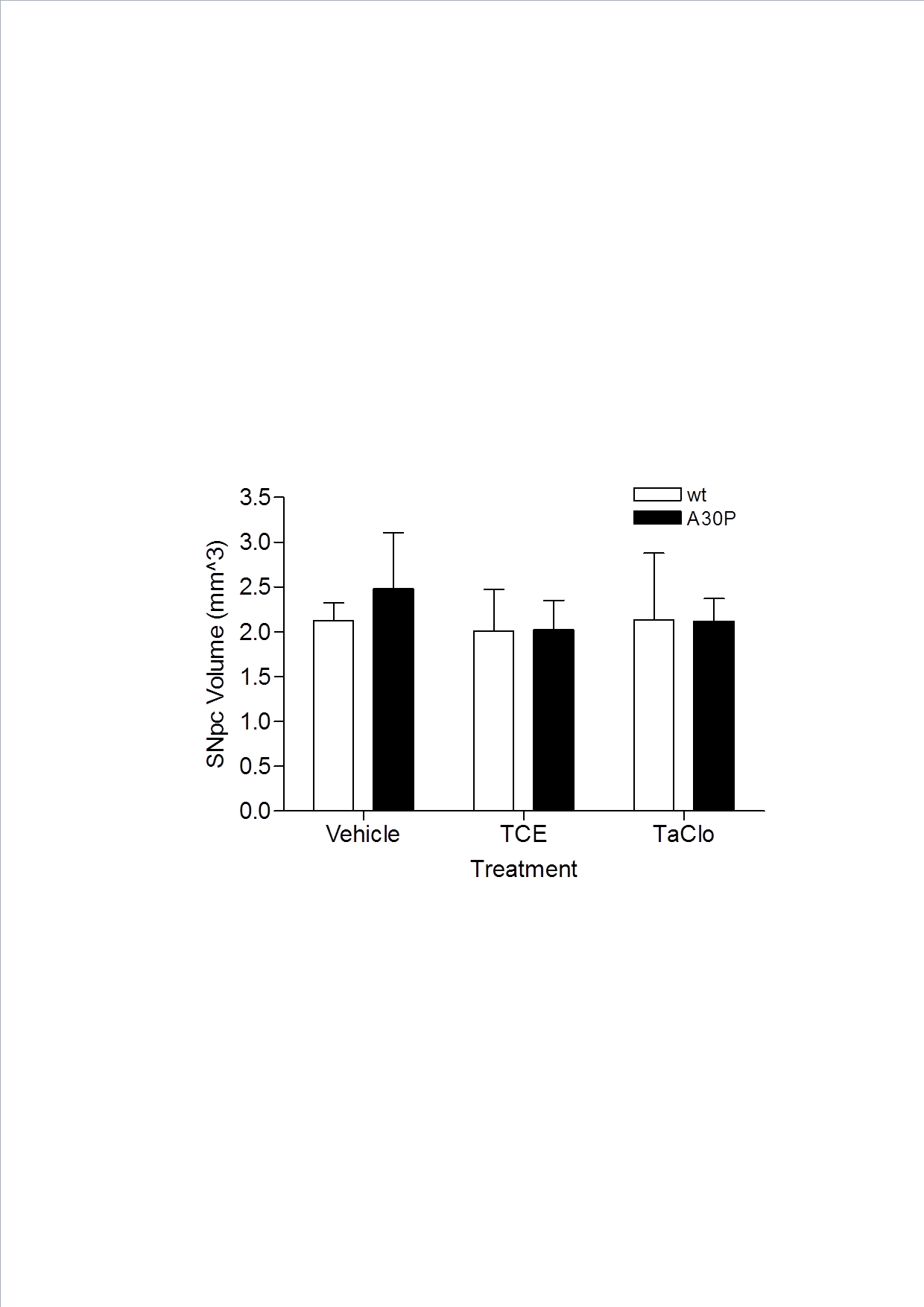


**Supplementary Fig. 5 SNpc volume in A30P α-synuclein overexpressing and wild type C57BL/6 Mice** Total SNpc sampling volume of TCE (1000mg/kg), TaClo (2mg/kg) and vehicle (olive oil) treated wild type (wt) and A30P overexpressing C57BL/6 mice assessed by stereological analysis. Data presented as mean ± SD (n=5/6). No significant difference between treatment groups or strains was observed.


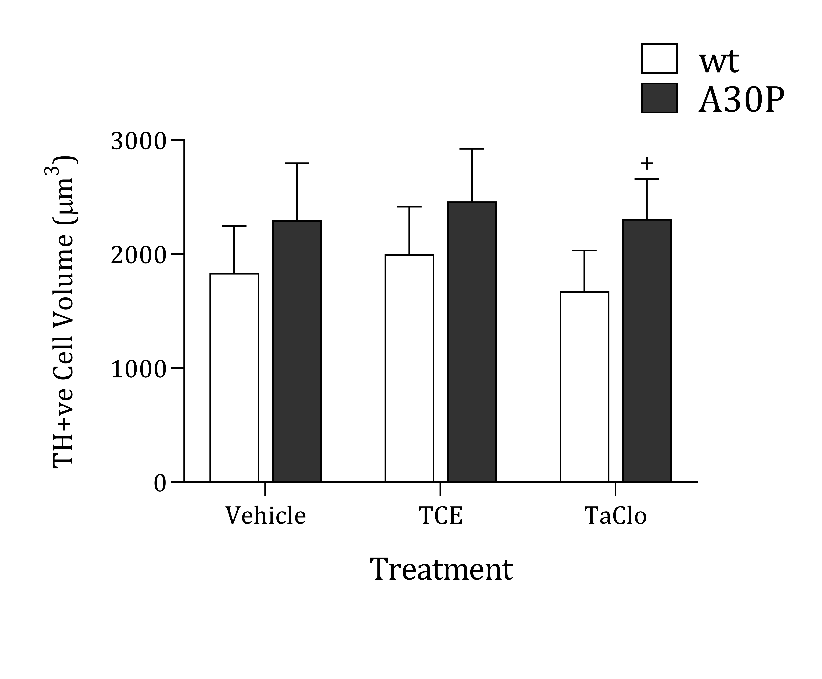


**Supplementary Fig. 6: Effect of TCE and TaClo on SNpc DA Neuron Volume in A30P α-synuclein overexpressing and wild type C57BL/6 Mice**

Cell body volume of TH-positive cells in the SNpc of TCE (1000mg/kg), TaClo (2mg/kg) and vehicle (olive oil) treated wild type (wt) and A30P overexpressing C57BL/6 mice assessed by stereological analysis. Data presented as mean + SD (n=5/6). +P<0.05 when compared to same treatment wt group, unpaired t-test.
